# Impact of the Oncologist's Recommendation on Exercise Levels and Quality of Life in Patients With Lung Cancer: The ORE Randomized Controlled Trial

**DOI:** 10.1002/cam4.71857

**Published:** 2026-04-26

**Authors:** Alice Avancini, Lorenzo Belluomini, Diana Giannarelli, Jessica Insolda, Anita Borsati, Marco Sposito, Jessica Menis, Paolo Lavagnolo, Daniela Tregnago, Ilaria Trestini, Federico Schena, Michele Milella, Sara Pilotto

**Affiliations:** ^1^ Section of Oncology, Department of Engineering for Innovation Medicine (DIMI) University of Verona and University and Hospital Trust (AOUI) of Verona Verona Italy; ^2^ Department of Neurosciences, Biomedicine and Movement Science University of Verona Verona Italy; ^3^ Fondazione Policlinico Universitario A. Gemelli IRCCS‐Epidemiology & Biostatistic Rome Italy; ^4^ Department of Medical Oncology University Hospital of Verona Verona Italy; ^5^ Dietetic Service, Medical Direction University Hospital of Verona (AOUI) Verona Italy

**Keywords:** behavior change, exercise recommendation, oncologist‐led intervention, physical exercise, quality of life

## Abstract

**Background:**

Although physical activity is associated with improved survival in lung cancer, most patients remain inactive. This study examined the impact of an oncologist's recommendation on exercise quantity, sedentary behavior, and quality of life (QoL) in patients with lung cancer.

**Methods:**

In this single‐blind, 3‐arm randomized controlled trial, patients with lung cancer (stages I–IV) were assigned to: (1) usual care, (2) the oncologist's verbal and written exercise recommendation, or (3) the recommendation plus a printed exercise guidebook. Exercise, sedentary behaviors, and QoL were assessed at baseline, 4, and 8 weeks. Descriptive statistics, analysis of variance, and a generalized linear regression model were used to analyze data.

**Results:**

Overall, 91 patients (*n* = 31 recommendation group, *n* = 31 recommendation + guidebook group, *n* = 29 controls) were randomized. At 8 weeks, the recommendation group showed a significant increase in total (+270 min/week, *p* = 0.03) and light‐intensity physical activity (+236 min/week, *p* = 0.01) versus controls. The recommendation plus guidebook group demonstrated a near‐significant increase in total (+251 min/week, *p* = 0.05) and moderate‐intensity physical activity (+145 min/week, *p* = 0.05). No significant changes were observed for sedentary behaviors. Regarding QoL, at 4 weeks, the recommendation plus guidebook group demonstrated an enhancement in cognitive function and the recommendation group in social function compared to controls. At 8 weeks, the recommendation + guidebook group reported improved constipation compared to the controls.

**Conclusions:**

A brief oncologist‐delivered recommendation effectively increased the total physical exercise in patients with lung cancer. Combining the oncologist's recommendation with a dedicated guidebook may be more effective in promoting moderate‐ and vigorous‐intensity exercise, although the short follow‐up limits conclusions regarding long‐term effects.

**Trial Registration:**

ClinicalTrial.gov identifier: NCT05497544

## 1 Introduction

Lung cancer remains a critical global burden of disease, accounting for approximately 2.48 million new cases and 1.81 million related deaths [1]. By 2050, these estimates are projected to rise to 4.62 million and 3.55 million, respectively [1]. Smoking is the main risk factor, but environmental exposures (e.g., arsenic, radon, air pollution) and unhealthy behaviors, such as being overweight and physically inactive, also contribute to risk [2, 3, 4]. Advances in early detection, diagnosis, and treatment have improved survival across disease stages. Nevertheless, patients continue to experience multiple symptoms and treatment‐related adverse events. More than 30% of treatment‐naïve patients report moderate to severe fatigue, sleep disturbance, distress, pain, dyspnea, sadness, or drowsiness [5], with symptom burden being especially pronounced in advanced disease [6, 7]. These symptoms negatively affect quality of life (QoL) and prognosis [6, 8], underscoring the need for supportive interventions.

Physical activity and exercise have been shown to be safe and effective strategies for mitigating these impairments and potentially improving outcomes. In the early stage setting, preoperative exercise can enhance cardiorespiratory fitness and muscular strength [9] while reducing postoperative complications and hospital stay [10]. During and after treatment, regular exercise improves physical function (e.g., fitness, strength, body composition) [11, 12] and helps manage side effects such as fatigue [13], sleep disorder [14], and anxiety/depression [15]. Epidemiological evidence indicates a survival benefit for physically active patients [16], and prognostic factors such as cardiorespiratory fitness, muscle mass, and strength are strongly influenced by exercise [17, 18, 19, 20, 21]. Preclinical studies also suggest that exercise may reduce tumor growth and synergize with anticancer therapies through multiple biological mechanisms [10].

Given these benefits, several societies, including the American Society of Clinical Oncology [22], the American Cancer Society [23], and the American College of Sports Medicine [24], recommend regular exercise for patients with cancer. Despite this, about 70% of patients with lung cancer remain insufficiently active [25]. Oncologists, as primary points of contact, can play a pivotal role in promoting physical activity through three simple steps: (i) identify inactive patients, (ii) explain the importance of activity during and after treatment, and propose suggestions according to guidelines, and (iii) refer patients to dedicated services [26, 27]. While assessment and advice are feasible and supported by validated tools, referral remains challenging due to the limited availability of programs [27, 28]. Previous studies in breast and colorectal cancer have shown that oncologist recommendations, whether verbal or written, can increase moderate‐intensity exercise and represent a cost‐ and time‐efficient strategy with potential clinical benefits [29, 30]. To date, however, no trials have investigated this approach in lung cancer. This study aimed to test the efficacy of two oncologist‐based interventions, recommendation alone and recommendation plus guidebook, on self‐reported physical exercise, sedentary behavior, and QoL in patients with lung cancer.

## 2 Materials and Methods

### 2.1 Study Design, Participants, and Procedures

The Oncologists Recommend Exercise in 30 s (O.R.E.—30 s) study is a single‐centre, single‐blind, three‐arm randomized controlled trial conducted at the University of Verona Hospital Trust to compare the impact of the oncologist's recommendations versus usual care on exercise levels in patients with lung cancer. The study was conducted in accordance with the principles of Good Clinical Practice and the ethical guidelines outlined in the Helsinki and Oviedo Declarations. Ethical approval was obtained from the Verona University Ethics Committee for Clinical Trials (Protocol No. 26622), and the study protocol was registered on ClinicalTrial.gov.

### 2.2 Eligibility Criteria, Procedures, and Randomization

Participants having a confirmed diagnosis of lung cancer (stages I–IV), age ≥ 18 years, an ECOG performance status of 0–1, and who signed the informed consent were eligible for study participation. Patients were ineligible if they had significant physical or psychological disabilities (e.g., wheelchair or psychiatric disorders) or if they were not able to speak, read, or write in the Italian language.

Patients were recruited between October 2022 and September 2024 at the Oncology Unit of the University of Verona Hospital Trust. Potential participants were screened for eligibility using electronic medical records before they arrived at the clinic. During routine check‐ups or day‐hospital visits for therapy, oncologists invited eligible patients to participate in the study. Those who agreed to participate and provided the written informed consent were assigned to one of three groups: (1) usual care, (2) oncologist's exercise recommendation, or (3) oncologist's exercise recommendation plus an exercise guidebook. During the initial study visit, research team members assisted participants in completing all required questionnaires. Participants were informed that they would be contacted by phone at 4 and 8 weeks for follow‐up assessments. Randomization (1:1:1 ratio) was stratified by cancer stage, with computer‐generated randomization lists for each stratum created using the permutation method with variable block sizes. The individual who coordinated the randomization process was not involved in any of the screening, intervention, or outcome procedures. Additionally, the blinding has been achieved through the concealment of the research assistant who performed the assessments at 4 and 8 weeks by telephone. The research assistant was trained to administer the telephone questionnaire using a standardized protocol, while the patients were instructed not to reveal their group allocation.

### 2.3 Intervention

Three oncologists received training, lasting approximately 90 min, with the research staff to overview study protocol and procedures, and to increase knowledge about exercise benefits and details regarding exercise recommendations and the guidebook. After baseline assessments, patients were randomly allocated to the oncologist's exercise recommendation group, the oncologist's exercise recommendation plus an exercise guidebook group, or the usual care group.

#### 2.3.1 Oncologist's Exercise Recommendation

This group received an oncologist's advice about exercise. The recommendation was both verbal and written in the clinical letter and included the following standardized statement based on current exercise guidelines for patients with cancer:

Preliminary evidence suggests that exercise may help to reinforce your immune system and potentially prevent cancer recurrence. Moreover, it may be a powerful tool to aid recovery from surgery and/or to manage some of the side effects you may experience during and after treatment. I suggest trying to exercise for at least 20–30 min, 4 or 5 days a week, at a moderate intensity, i.e., an intensity at which you can speak but not sing. Even less may be beneficial; you can try brisk walking, for example.

#### 2.3.2 Oncologist's Exercise Recommendation Plus an Exercise Guidebook

In addition to receiving verbal and written information, a printed exercise manual, called “Informa”, specifically designed for patients with cancer, was distributed to this group by the oncologist. The guidebook was developed through a multi‐step process and was informed by the Theory of Planned Behavior. This process included team discussions to define aims and target population, an in‐depth literature review on exercise‐oncology, and the integration of evidence from previous studies on patient preferences and barriers to physical activity. Drafting was theory‐driven and oriented by recommendations for suitability and readability, followed by iterative revisions and consensus within the research team. Finally, the guidebook was pilot‐tested to assess readability and suitability before its use [31]. The manual includes 11 chapters that cover key aspects of exercise in oncology, including benefits, evidence‐based recommendations, and instructions for achieving and monitoring exercise intensity, safety precautions, and practical strategies for incorporating exercise into daily life.

#### 2.3.3 Usual Care

The control group received standard oncological care. After the study's completion, participants in the control group received the advice and exercise guidebook.

### 2.4 Endpoints

#### 2.4.1 Primary Endpoint

The primary endpoint was the change in self‐reported total exercise amount from baseline to 4 weeks. Physical exercise levels were assessed using the Godin‐Shephard Leisure‐Time Exercise Questionnaire [32], which records the frequency (days per week) and duration (minutes per day) of light, moderate, and vigorous exercise. By multiplying frequency and duration, the overall minutes per week for each intensity level were calculated. Then, by summing the minutes for each intensity, the total weekly time spent in exercise was obtained [32].

#### 2.4.2 Secondary Endpoints

Secondary endpoints included changes in exercise amount at 8 weeks, as well as sedentary behaviors and QoL at 4 and 8 weeks. Sedentary behaviors were assessed using the validated Sedentary Behavior Questionnaire. This questionnaire is composed of 18 items assessing the hours spent in different sedentary activities such as watching television, playing computer/video games, sitting while listening to music, sitting and talking on the phone, doing paperwork or office work, sitting and reading, playing a musical instrument, doing arts and crafts, sitting and driving/riding in a car, bus, train. The questionnaire assessed sedentary behavior on weekdays and weekends [33].

The QoL was measured using the European Organization for Research and Treatment of Cancer Quality of Life Questionnaire (EORTC QLQ‐C30). This 30‐item questionnaire comprises multi‐item scales and single items that reflect the multidimensionality of the QoL construct. It incorporates five functional scales (physical, role, cognitive, emotional, and social), three symptom scales (fatigue, pain, and nausea and vomiting), and a global health and QoL scale. The remaining single items assess additional symptoms commonly reported by cancer patients (dyspnea, appetite loss, sleep disturbance, constipation, and diarrhea) [34].

At baseline, demographic information, including age, gender, education, marital status, and employment, was collected through a self‐report questionnaire. Clinical data, including tumor type, stage of disease, cancer treatments, comorbidities, and smoking status, were extracted from medical charts.

### 2.5 Statistical Considerations

#### 2.5.1 Sample Size Calculation

For sample size calculation, we assumed the total exercise amount value from a previous similar study [29]. We set the minimum detectable difference in the mean to 40 min per week of physical exercise, with an expected standard deviation of 40 among experimental groups versus controls, a power of 0.95, and an alpha level of 0.05. A total of 26 patients were requested per group. Considering a possible dropout rate of 18%, a total of 30 patients per group were recruited.

#### 2.5.2 Statistical Analysis

Descriptive statistics were applied to demographic and clinical data. Mean values of outcomes, along with their 95% confidence intervals, were reported. The repeated‐measures analysis of variance was applied to assess within‐group differences over time and to investigate unadjusted comparisons between the three groups. The generalized linear regression model analysis was used to compare the differences across groups in changes over time, adjusting the between‐groups differences for baseline outcome data, age, sex, body mass index, smoking status, education, marital status, and income status. The intention‐to‐treat principle was used to analyze data, and statistical significance was defined as *p* < 0.05, two‐sided. No adjustment for multiple testing was applied. All analyses were performed using IBM SPSS Statistics (version 28.0; IBM Corporation, New York, NY).

## 3 Results

**FIGURE 1:**
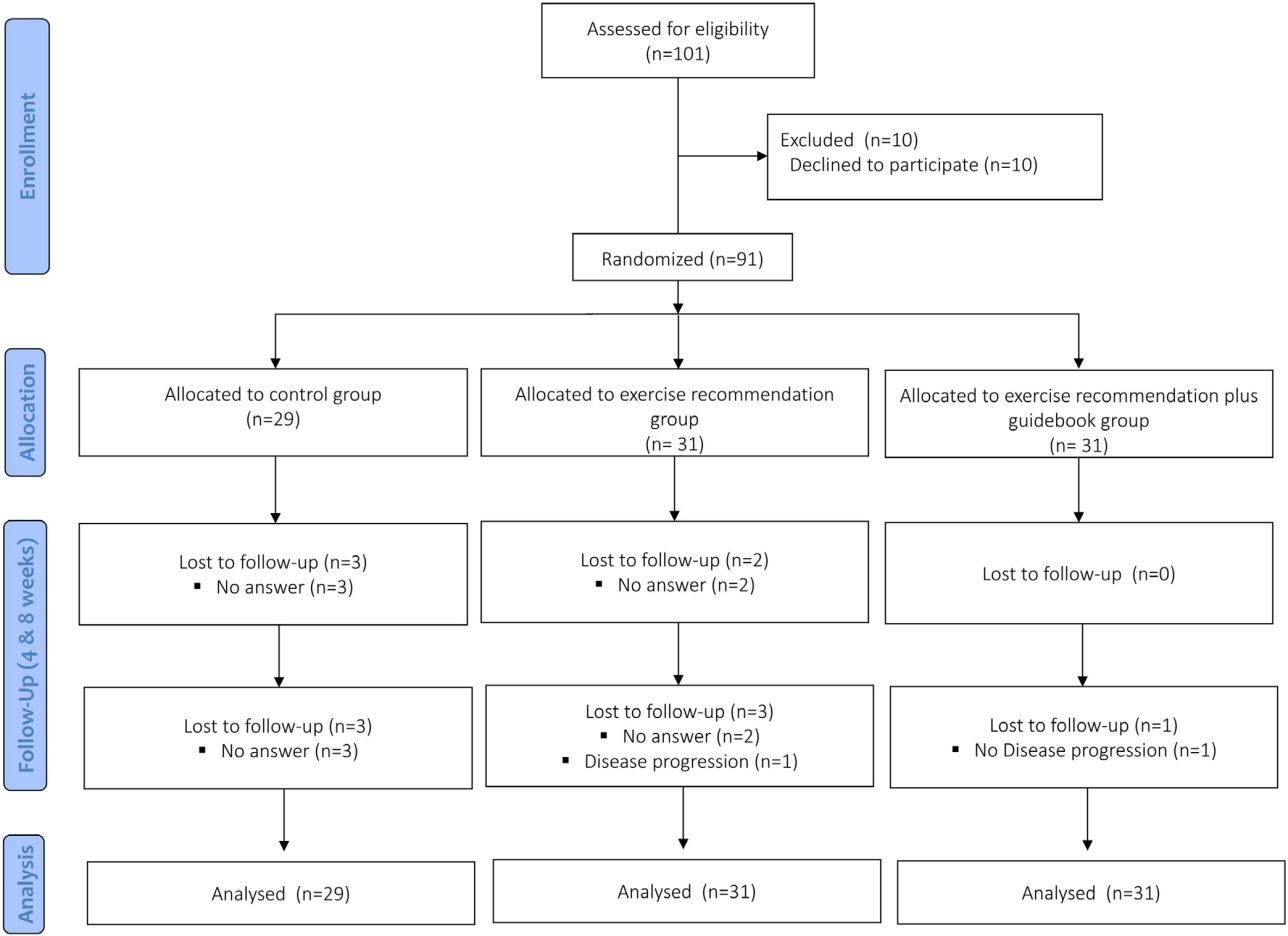
Flow of participants through the study.

Overall, 101 patients with lung cancer were screened for eligibility. Ten participants declined to participate, and 91 were randomized. A total of 84 patients (92%) completed the study (Figure 1).

The median age of the participants was 67 years old; 50% were male, 71% were married, and 57% were retired. About 70% of patients were current or former smokers, 86% had a non‐small cell lung cancer, and 70% had a stage IV disease. The median time since diagnosis was 3 months, and 86% of participants presented comorbidities. The three groups were balanced in terms of demographics and medical characteristics (Table 1).

**TABLE 1:** Baseline demographic and medical profile of participants overall and by group assignment.

Characteristics	Overall (*n* = 91)	Control (*n* = 29)	Exercise recommendation (*n* = 31)	Exercise recommendation plus guidebook (*n* = 31)
Demographics				
Age, median (IQR)	67 (59–72)	65 (59–72)	66 (59–72)	69 (62–73)
Gender, *n* (%)				
Male	46 (50.5)	16 (55.2)	16 (51.6)	14 (45.2)
Female	45 (49.5)	13 (44.8)	15 (48.4)	17 (54.8)
Marital status, *n* (%)				
Married	65 (71.4)	23 (79.3)	20 (64.5)	22 (71.0)
Single	8 (8.8)	3 (10.3)	3 (9.7)	2 (6.5)
Divorced	7 (7.7)	2 (6.9)	2 (6.5)	3 (9.7)
Widow	11 (12.1)	1 (3.4)	6 (19.4)	4 (12.9)
Education, *n* (%)				
Primary	18 (19.8)	4 (13.8)	6 (19.4)	8 (25.8)
Secondary	34 (37.4)	15 (51.7)	10 (32.3)	9 (29.0)
High school degree	28 (30.8)	7 (24.1)	12 (38.7)	9 (29.0)
Undergraduate degree	11 (12.1)	3 (10.3)	3 (9.7)	5 (16.1)
Employment, *n* (%)				
Full‐time employed	25 (27.5)	9 (31.0)	7 (22.6)	9 (29.0)
Part‐time employed	3 (3.3)	1 (3.4)	1 (3.2)	1 (3.2)
Retired	52 (57.1)	16 (55.2)	18 (58.1)	18 (58.1)
Unemployed	5 (5.5)	1 (3.4)	3 (9.7)	1 (3.2)
Homemaker	6 (6.6)	2 (6.9)	2 (6.5)	2 (6.5)
Family income, *n* (%)				
Inadequate	3 (3.3)	1 (3.4)	1 (3.2)	1 (3.2)
Barely adequate	15 (16.5)	4 (13.8)	7 (22.6)	4 (12.9)
Adequate	60 (65.9)	20 (69.0)	19 (61.3)	21 (67.7)
More than adequate	13 (14.3)	4 (13.8)	4 (12.9)	5 (16.1)
Medical information				
BMI, median (IQR)	24.1 (22.0–26.7)	24.3 (23.2–26.6)	23.8 (20.4–27.8)	23.9 (20.4–27.8)
Smoking habits, *n* (%)				
Never smoker	27 (29.7)	3 (10.3)	9 (29.0)	15 (48.4)
Former smoker	45 (49.5)	19 (65.5)	15 (48.4)	11 (35.5)
Current smoker	19 (20.9)	7 (24.1)	7 (22.6)	5 (16.1)
Histology, *n* (%)				
Non‐small cell	78 (85.7)	24 (82.7)	25 (80.7)	29 (93.5)
Small cell	10 (11.0)	4 (13.8)	5 (16.1)	1 (3.2)
Other	3 (3.3)	1 (3.4)	1 (3.2)	1 (3.2)
Stage, *n* (%)				
I	12 (13.2)	5 (17.2)	4 (12.9)	3 (9.7)
II	12 (13.2)	3 (10.3)	4 (12.9)	5 (16.1)
III	3 (3.3)	0 (0.0)	1 (3.2)	2 (6.5)
IV	64 (70.3)	21 (72.4)	22 (71.0)	21 (67.7)
Treatments, *n* (%)				
Surgery	48 (52.7)	14 (48.3)	16 (51.6)	18 (58.1)
Radiotherapy	5 (5.5)	0 (0.0)	3 (9.7)	2 (6.5)
Chemotherapy	33 (36.3)	11 (37.9)	10 (32.3)	12 (38.7)
Target therapy	23 (25.3)	5 (5.5)	11 (35.5)	7 (22.6)
Immunotherapy	35 (38.5)	10 (34.5)	13 (41.9)	12 (38.7)
Months since diagnosis, median (IQR)	3 (1–10)	2 (1–10)	7 (2–10.5)	3 (1.5–10)
Comorbidities, *n* (%)				
Yes	78 (85.7)	26 (89.7)	25 (80.6)	27 (87.1)
No	13 (14.3)	3 (10.3)	6 (19.4)	4 (12.9)

Abbreviations: %, percentage; IQR, interquartile range; *n*, number.

### 3.1 Effect on Physical Exercise Levels

Table 2 presents the adjusted outcomes related to physical exercise levels. At 4 weeks, within‐group analyses showed that the control group increased total exercise by +175 min/week (95% CI: +29.0; +321.4), while the exercise recommendation group gained +138 min/week (95% CI: −119.9; +396.2), and the guidebook group declined by −109 min/week (95% CI: −376.8; +158.2). However, between‐group comparisons did not reveal statistically significant differences. At 8 weeks, compared with controls, the exercise recommendation group increased total activity by +270 min/week (95% CI: +22.2; +518.3; *p* < 0.05) and light activity by +236 min/week (95% CI: +45.6; +425.7; p < 0.05). Clinically, this increment is meaningful, as although the intensity was not moderate, it exceeds the 150 min/week threshold recommended by international guidelines for patients with cancer. However, moderate and vigorous activities also increased, though without statistical significance. The exercise recommendation plus guidebook group demonstrated near‐significant improvements compared with controls in both total (+251 min/week; 95% CI: −7.6; +508.7; *p* = 0.05) and moderate‐intensity exercise (+145 min/week; 95% CI: −0.8; +291.2; p = 0.05). Although borderline in statistical terms, these increases may be considered clinically relevant. As no validated cutoff for clinically meaningful change exists for the Godin Leisure‐Time Exercise Questionnaire, and given baseline activity levels of approximately 40 min per week, an additional 145 min would place participants above the commonly recommended 150‐min‐per‐week threshold. This value was therefore used as a pragmatic reference for clinical interpretation. Compared with the recommendation‐only group, the guidebook group showed a non‐significant decline in light activity but higher gains in moderate and vigorous activity, suggesting a possible shift in exercise intensity. In the Supporting Information, unadjusted analyses are presented.

**TABLE 2:** Effect of the oncologist's recommendations on physical exercise levels in patients with lung cancer.

	Within‐group differences			Between‐group differences^a^		
	Control	Exercise recommendation	Exercise recommendation plus guidebook	Exercise recommendation vs. control	Exercise recommendation plus guidebook vs. control	Exercise recommendation plus guidebook vs. exercise recommendation
	Mean (95% CI)	Mean (95% CI)	Mean (95% CI)	Mean (95% CI)	Mean (95% CI)	Mean (95% CI)
Total (min/week)						
Baseline	108.8 (+42.1; +175.5)	223.2 (+140.5; +305.9)	252.6 (+0.4; +504.7)			
Change from 0 to 4 weeks	+175.2 (+29.0; +321.4)	+138.2 (−119.9; +396.2)	−109.3 (−376.8; +158.2)	+112.9 (−107.2; +333.1)	−55.8 (−286.5; +174.9)	−168.7 (−382.9; +45.5)
Change from 0 to 8 weeks	+46.7 (−18.9; +112.3)	+157.1 (−49.4; +363.7)	+45.3 (−323.3; +413.9)	+270.2 (+22.2; +518.3)*	+250.6 (−7.6; +508.7)^#^	−19.7 (−262.8; +223.4)
Vigorous (min/week)						
Baseline	0.0 (0.0; 0.0)	1.9 (0.0; +5.9)	0.0 (0.0; 0.0)			
Change from 0 to 4 weeks	0.0 (0.0; 0.0)	+10.7 (−11.3; +32.7)	+1.0 (−1.1; +3.2)	+11.6 (−5.1; +28.4)	−2.5 (−19.9; +14.9)	−14.2 (−30.5; +2.2)
Change from 0 to 8 weeks	0.0 (0.0; 0.0)	+8.1 (−8.6; +24.7)	+12.0 (−12.5; +36.5)	+10.8 (−12.5; +34.1)	+18.3 (−5.4; +42.1)	+7.5 (−15.2; +30.3)
Moderate (min/week)						
Baseline	11.7 (0; 26.0)	41.8 (4.0; 79.5)	12.4 (1.5; 23.3)			
Change from 0 to 4 weeks	+12.8 (−18.4; +44.0)	−35.2 (−79.0; +8.6)	+17.1 (−11.0; +45.1)	−15.9 (−47.3; +15.4)	+5.7 (−26.1; +37.6)	+21.7 (−9.1; +52.5)
Change from 0 to 8 weeks	+23.4 (−6.0; +52.8)	−0.8 (−51.2; +49.7)	+119.0 (−42.5; +280.5)	+4.2 (−143.3; +151.7)	+145.2 (−0.8; +291.2)^#^	+141.0 (−3.3; +285.3)
Light (min/week)						
Baseline	97.1 (+38.4; +155.8)	179.5 (+109.6; +249.4)	240.2 (0.0; +486.6)			
Change from 0 to 4 weeks	+162.4 (+17.5; +307.3)	+162.7 (−82.6; +407.9)	−127.4 (−388.9; +134.1)	+114.7 (−99.7; +329.0)	−59.3 (−285.1; +166.4)	−174.0 (−384.0; +35.9)
Change from 0 to 8 weeks	+57.6 (−0.3; +115.5)	+222.5 (+6.2; +438.8)	−83.5 (−362.4; +195.4)	+235.7 (+45.6; +425.7)*	+63.0 (−132.4; +258.4)	−172.6 (−359.2; +13.9)

^a^ Values adjusted for baseline outcome data, age, sex, body mass index, smoking status, education, marital status, and income status.

* *p* < 0.05.

^#^ *p* = 0.05.

### 3.2 Effect on Sedentary Behaviors and Quality of Life

The effects of interventions on sedentary behaviors and QoL were listed in Table 3. For sedentary behaviors, within‐group analyses showed small reductions in the controls (−4.4 h/week) and the guidebook group (−5.5 h/week) at 8 weeks, while the recommendation group increased slightly (+3.7 h/week). Between‐group differences were not statistically significant, although both intervention arms showed trends toward less sedentary time compared with controls.

**TABLE 3:** Effect of the oncologist's recommendations on sedentary behaviors and quality of life in patients with lung cancer.

	Within‐group differences			Between group differences^a^		
	Control	Exercise recommendation	Exercise recommendation plus guidebook	Exercise recommendation vs. control	Exercise recommendation plus guidebook vs. control	Exercise recommendation plus guidebook vs. exercise recommendation
	Mean (95% CI)	Mean (95% CI)	Mean (95% CI)	Mean (95% CI)	Mean (95% CI)	Mean (95% CI)
Total sedentary behaviors (hour/week)						
Baseline	41.9 (33.3; 50.5)	35.6 (29.4; 41.8)	47.1 (40.2; 54.0)			
Change from 0 to 4 weeks	−4.1 (−14.4; 6.3)	2.6 (−3.2; 8.3)	−8.6 (−16.3; −0.8)	−2.4 (−10.0; 5.1)	0.1 (−7.6; 7.9)	2.6 (−4.9;10.11)
Change from 0 to 8 weeks	−4.4 (−11.5; 2.6)	3.7 (−2.5; 10.0)	−5.5 (−14.2; 3.3)	−5.1 (−13.2; 3.1)	−3.7 (−12.0; 4.5)	1.3 (−6.9; 9.5)
Sedentary behaviors weekday (hour/day)						
Baseline	6.2 (4.9; 7.6)	5.3 (4.3; 6.3)	7.2 (6.0; 8.3)			
Change from 0 to 4 weeks	−0.9 (−2.5; 0.7)	0.2 (−0.7; 1.2)	−1.5 (−2.8; −0.2)	−0.4 (−1.6; 0.7)	−0.2 (−1.5; 1.0)	0.2 (−1.0; 1.4)
Change from 0 to 8 weeks	−0.9 (−2.0; 0.2)	0.4 (−0.6; 1.5)	−1.2 (−2.7; 0.2)	−0.7 (−2.0; 0.5)	−0.4 (−1.7; 0.8)	0.3 (−0.9; 1.6)
Sedentary behaviors weekend (hour/day)						
Baseline	5.4 (4.1; 6.7)	4.6 (3.7; 5.5)	5.6 (4.7; 6.6)			
Change from 0 to 4 weeks	0.2 (−1.4; 1.9)	0.8 (−0.1; 1.6)	−0.5 (−1.4; 0.5)	0.1 (−0.99; 1.1)	0.6 (−0.5; 1.6)	0.5 (−0.5; 1.5)
Change from 0 to 8 weeks	−0.1 (−1.1; 1.0)	0.7 (−0.1; 1.5)	0.3 (−0.9; 1.6)	−0.4 (−1.6; 0.8)	−1.0 (−2.2; 0.2)	−0.5 (−1.7; 0.7)
Quality of life (EORTC‐QLQ‐C30)						
Physical function						
Baseline	81.4 (74.3; 88.6)	84.5 (78.2; 90.9)	83.2 (76.6; 89.9)			
Change from 0 to 4 weeks	1.6 (−5.6; 8.8)	3.3 (−1.7; 8.3)	3.2 (−3.6; 10.0)	−3.1 (−9.4; 3.2)	−0.6 (−7.1; 6.0)	2.5 (−3.6; 8.7)
Change from 0 to 8 weeks	6.1 (−2.3; 14.5)	−1.5 (−6.6; 3.5)	1.8 (−6.0; 9.6)	5.2 (−2.0; 12.3)	3.0 (−4.2; 10.2)	−2.2 (−9.2; 4.8)
Role function						
Baseline	80.9 (70.6; 91.3)	77.9 (67.0; 88.9)	83.3 (75.1; 91.5)			
Change from 0 to 4 weeks	4.7 (−10.8; 20.1)	5.9 (−4.0; 15.9)	−5.7 (−17.3; 5.8)	1.2 (−13.1; 15.5)	9.7 (−5.2; 24.7)	8.6 (−5.5; 22.7)
Change from 0 to 8 weeks	8.0 (0.3; 16.0)	6.4 (−2.3; 15.1)	−2.2 (−13.7; 9.3)	1.1 (−12.9; 15.1)	5.7 (−8.5; 19.9)	4.6 (−9.1; 18.1)
Emotional function						
Baseline	72.0 (62.9; 81.1)	76.3 (68.2; 84.5)	68.3 (57.9; 78.7)			
Change from 0 to 4 weeks	8.3 (−0.2; 16.8)	2.1 (−4.4; 8.5)	12.9 (2.3; 23.6)	3.1 (−5.4; 11.6)	2.1 (−6.7; 11.0)	−1.0 (−9.4; 7.3)
Change from 0 to 8 weeks	10.3 (−0.5; 21.1)	4.5 (−3.6; 12.6)	11.1 (0.1; 22.1)	2.1 (−6.9; 11.1)	3.0 (−6.2; 12.1)	0.8 (−8.0; 9.7)
Cognitive function						
Baseline	88.7 (81.0; 96.4)	88.7 (82.9; 94.5)	90.9 (85.9; 95.8)			
Change from 0 to 4 weeks	9.3 (1.6; 17.0)	0.6 (−8.8; 10.0)	−2.3 (−7.6; 3.0)	7.1 (−0.8; 15.0)	10.1 (1.8; 18.3)*	3.0 (−4.7; 10.8)
Change from 0 to 8 weeks	4.7 (−3.9; 13.2)	−1.9 (−8.6; 4.8)	−1.7 (−5.4; 2.1)	3.3 (−3.8; 10.5)	4.8 (−2.5; 12.0)	1.4 (−5.6; 8.5)
Social function						
Baseline	82.1 (73.4; 90.9)	84.9 (76.4; 93.5)	83.3 (75.6; 91.1)			
Change from 0 to 4 weeks	−2.0 (−14.0; 10.0)	1.8 (−5.1; 8.7)	−10.9 (−20.7; −1.1)	−7.3 (−19.6; 5.1)	7.9 (−5.0; 20.7)	15.1 (3.0; 27.3)*
Change from 0 to 8 weeks	1.3 (−8.8; 11.4)	−3.2 (−13.6; 7.2)	−5.6 (−13.6; 2.5)	−0.5 (−11.5; 10.5)	2.7 (−8.5; 13.9)	3.2 (−7.6; 14.0)
Global health status						
Baseline	66.1 (57.0; 75.2)	72.3 (65.3; 79.3)	70.2 (62.0; 78.3)			
Change from 0 to 4 weeks	0 (−10.8; 10.8)	2.4 (−6.0; 10.8)	−2.6 (−10.1; 4.9)	−8.3 (−19.8; 3.2)	−2.3 (−14.2; 9.7)	6.0 (−5.2; 17.3)
Change from 0 to 8 weeks	5.3 (−5.9; 16.5)	7.1 (−3.8; 17.9)	−2.8 (−12.0; 6.4)	−8.1 (−18.4; 2.2)	0.3 (−10.1; 10.8)	8.4 (−1.7; 18.5)
Fatigue						
Baseline	27.8 (17.6; 38.0)	24.7 (16.3; 33.2)	22.2 (12.9; 31.6)			
Change from 0 to 4 weeks	3.6 (−9.7; 16.8)	−4.4 (−14.5; 5.8)	−1.1 (−9.0; 6.7)	10.6 (−0.6; 21.8)	6.6 (−5.1; 18.2)	−4.0 (−15.0; 6.9)
Change from 0 to 8 weeks	−8.9 (−5.8; 10.2)	2.1 (−10.1; 14.4)	2.2 (−5.8; 10.3)	−9.5 (−20.2; 1.2)	−9.3 (−20.2; 1.6)	0.2 (−10.2; 10.7)
Nausea vomiting						
Baseline	4.8 (0.0; 9.7)	4.8 (1.2; 8.4)	3.2 (0.3; 6.1)			
Change from 0 to 4 weeks	4.0 (−3.5; 11.5)	4.2 (−1.3; 9.6)	5.2 (−1.0; 11.3)	−0.9 (−9.1; 7.2)	−3.9 (−12.4; 4.5)	−3.0 (−11.0; 4.9)
Change from 0 to 8 weeks	3.3 (−2.9; 9.6)	4.5 (−0.4; 9.4)	5.0 (−0.9; 10.9)	−1.0 (−8.5; 6.6)	−0.2 (−7.9; 7.5)	−0.8 (−6.6; 8.2)
Pain						
Baseline	25.0 (4.5; 35.5)	24.2 (15.7; 32.7)	19.4 (10.3; 28.4)			
Change from 0 to 4 weeks	−6.0 (−13.9; 1.9)	−4.8 (12.6; 3.1)	−6.9 (−17.5; 3.7)	1.4 (−10.9; 13.7)	3.4 (−9.4; 16.2)	2.0 (−10.0; 14.0)
Change from 0 to 8 weeks	−12.0 (−22.0; −2.0)	−2.6 (−9.6; 4.5)	−6.7 (−16.8; 3.5)	−5.9 (−16.2; 4.4)	0.3 (−10.2; 10.8)	6.2 (−3.9; 16.3)
Dyspnea						
Baseline	29.8 (20.2; 39.3)	23.7 (12.6; 34.7)	30.1 (19.9; 40.3)			
Change from 0 to 4 weeks	−6.7 (−17.2; 3.8)	−1.2 (−14.1; 11.7)	−8.0 (−21.9; −7.1)	−3.0 (−15.9; 9.8)	−1.3 (−14.7; 12.1)	1.7 (−10.9; 14.3)
Change from 0 to 8 weeks	−12.0 (−23.8; −0.2)	−2.6 (−13.9; 8.8)	−7.8 (−19.4; 3.9)	−6.6 (−19.7; 6.6)	−8.5 (−21.9; 4.8)	−2.0 (−14.8; 10.9)
Insomnia						
Baseline	29.8 (18.5; 41.1)	20.4 (10.1; 30.7)	23.7 (12.2; 35.1)			
Change from 0 to 4 weeks	−1.3 (−15.4; 12.7)	−4.8 (−11.5; 2.0)	−5.7 (−18.9; 7.4)	9.2 (−4.6; 23.1)	5.4 (−9.0; 19.8)	−3.8 (−17.4; 9.7)
Change from 0 to 8 weeks	−2.7 (−14.5; 9.2)	0.0 (−6.6; 6.6)	−6.7 (−18.2; 4.8)	1.9 (−13.0; 16.7)	9.5 (−5.7; 24.6)	7.7 (−6.8; 22.2)
Appetite loss						
Baseline	13.1 (3.6; 22.6)	12.9 (3.6; 22.2)	12.9 (4.2; 21.6)			
Change from 0 to 4 weeks	−4.0 (−14.0; 6.0)	−7.1 (−17.9; 3.6)	2.3 (−10.3; 14.9)	2.8 (−9.6; 15.1)	−8.1 (−20.9; 4.8)	−10.8 (−22.9; 1.2)
Change from 0 to 8 weeks	−2.7 (−14.5; 9.2)	−2.6 (−12.6; 7.5)	2.2 (−10.4; 14.8)	1.7 (−9.8; 13.3)	0.3 (−11.4; 12.0)	−1.4 (−12.7; 9.8)
Constipation						
Baseline	22.6 (11.5; 33.8)	16.1 (6.7; 25.5)	19.3 (8.5; 30.2)			
Change from 0 to 4 weeks	2.7 (−8.5; 13.8)	−1.2 (−7.7; 5.4)	5.7 (−6.0; 17.5)	12.2 (−2.9; 27.4)	1.7 (−14.2; 17.5)	−10.6 (−25.5; 4.3)
Change from 0 to 8 weeks	−14.7 (−28.5; −0.9)	2.6 (−7.5; 12.6)	2.2 (−7.5; 12.0)	−8.7 (−21.5; 4.1)	−14.8 (−27.9; −1.8)*	−6.2 (−18.7; 6.3)
Diarreha						
Baseline	7.1 (0.0; 14.5)	7.5 (1.4; 13.6)	8.6 (1.6; 15.6)			
Change from 0 to 4 weeks	−1.3 (−14.2; 11.5)	−1.2 (−7.7; 5.4)	−3.4 (−14.3; 7.5)	−0.5 (−10.1; 9.0)	0.4 (−9.5; 10.3)	0.9 (−8.4; 10.3)
Change from 0 to 8 weeks	−2.7 (−11.5; 6.1)	0.0 (−8.5; 8.5)	5.6 (−3.1; 14.3)	−2.7 (−13.2; 7.7)	−6.1 (−16.8; 4.5)	−3.4 (−13.6; 6.8)
Financial problems						
Baseline	13.1 (4.2; 21.9)	10.7 (1.6; 19.9)	11.8 (5.1; 18.6)			
Change from 0 to 4 weeks	4.0 (−6.7; 14.7)	4.8 (−3.6; 13.2)	11.5 (0.6; 22.4)	3.9 (−9.6; 17.4)	−7.5 (−21.6; 6.5)	−11.4 (−24.6; 1.8)
Change from 0 to 8 weeks	2.7 (−7.0; 12.3)	2.6 (−7.5; 12.6)	4.4 (−4.6; 13.5)	4.8 (−6.1; 15.8)	−2.0 (−13.1; 9.2)	−6.8 (−17.5; 4.0)

^a^ Values adjusted for baseline outcome data, age, sex, body mass index, smoking status, education, marital status, and income status.

* *p* < 0.05.

Regarding QoL, most changes did not reach statistical significance. However, at 4 weeks, the exercise recommendation plus guidebook group showed a significant between‐group improvement in cognitive function versus controls (+10.1 points; 95% CI: +1.8; +18.3; *p* < 0.05). This exceeds the 10‐point threshold generally considered clinically meaningful for EORTC QLQ‐C30 scales [32]. Additionally, the exercise recommendation plus guidebook group reported better social function compared with the recommendation group (+15.1 points; 95% CI: +3.0; +27.3; *p* < 0.05), again surpassing the clinically meaningful threshold. These changes did not persist at 8 weeks. At 8 weeks, the exercise recommendation plus guidebook group reported significantly less constipation compared with controls (−14.8 points; 95% CI: −27.9; −1.8; *p* < 0.05), also exceeding the threshold for clinical relevance. Other symptoms and global health scales showed small, non‐significant changes across groups. In the Supporting Information, unadjusted analyses for sedentary behaviors and QoL are presented.

## 4 Discussion

**FIGURE 2:**
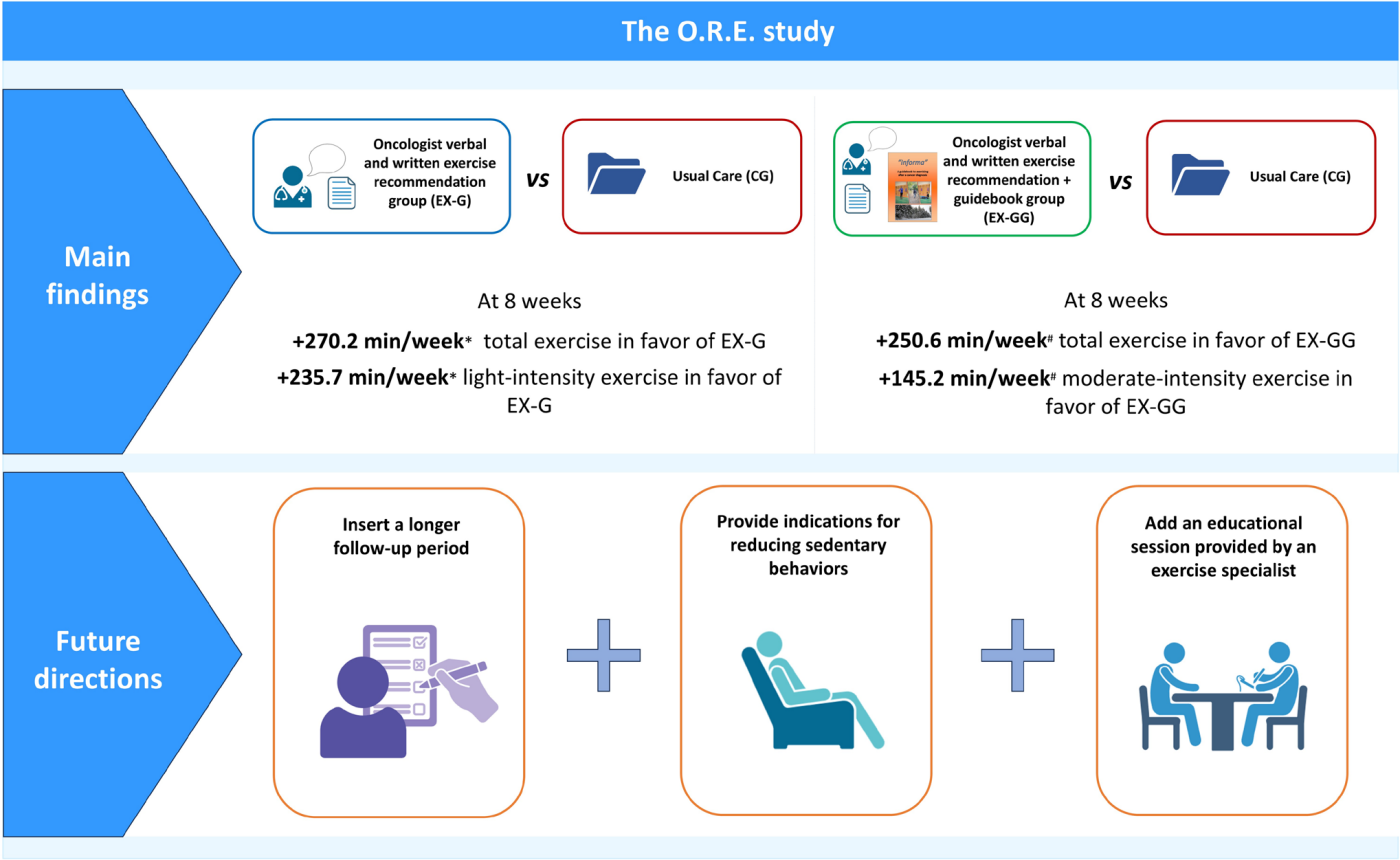
Main results and future directions of the O.R.E. study. Data are presented as mean differences in minutes/week (95% CI). Sample sizes were EX‐G (*n* = 31), EX‐GG (*n* = 31), and CG (*n* = 29). Between‐group comparisons were performed using generalized linear regression models adjusted for baseline values. *p < 0.05; ^#^ *p* = 0.05; ns, not significant.

This randomized controlled trial demonstrates that a simple oncologist‐delivered recommendation significantly increases physical activity levels in patients with lung cancer. To our knowledge, this is the first study to test the efficacy of a brief, easily implementable strategy, delivered by the treating oncologists, on exercise behavior in this population, including those with advanced‐stage disease (Figure 2).

We found that the oncologist's recommendations, provided both verbally and in the hospital discharge letter, resulted in a significant increase of approximately 270 min per week of physical exercise compared to the usual care approach. This finding suggests that patients are highly sensitive to the oncologist's advice, further reinforcing the existing literature that supports the central role of clinicians in patient care. However, the improvements occurred only for light‐intensity activities. The current exercise recommendations, by starting slowly and progressively increasing, recommend that patients engage in at least moderate‐intensity exercise. This is because low‐intensity exercise has been demonstrated to confer minimal benefits for physical fitness and symptom management compared to higher intensities [35, 36, 37]. Nevertheless, the information provided by the oncologist offered few details regarding the monitoring modalities and the progression to achieve the right intensity during exercise. In this sense, adding more comprehensive instructions may be relevant. Notably, the group receiving both the oncologist's recommendation plus a dedicated guidebook, which deeply explained how to reach and monitor the target intensity, also reported a gain near statistical significance in total exercise, predominantly at the moderate‐intensity level. Indeed, compared to controls, the exercise recommendation plus guidebook group increased by 145 min per week moderate‐intensity exercise, bringing participants above the commonly recommended 150‐min‐per‐week threshold [38]. Prior investigations have evaluated oncologist‐based interventions, finding mixed results. A first randomized trial on patients with early‐stage breast cancer attending adjuvant therapy found that the oncologist's verbal recommendation was able to increase by 30 min per week of moderate intensity at 5 weeks post‐consultation [29]. A second study, conducted on patients affected by early‐stage breast or colorectal cancers that had completed primary adjuvant treatments, observed a significant increase in moderate‐intensity and total exercise levels in patients who received the oncologist's recommendation with an exercise motivation package compared to controls after 4 weeks. Instead, no changes were detected in the group receiving the sole recommendation [30]. However, our study included a different population. Patients with lung cancer usually present more symptoms compared to those having other cancer types, such as colorectal tumors [39]. Additionally, in our cohort, most of the patients had metastatic lung cancer, which, unfortunately, has a dismal prognosis compared to early‐stage breast and colorectal cancers. In this light, our findings are even more encouraging, considering the association between physical activity and lung cancer survival [16], and suggest that, also in a population in which physical activity could be more complex to practice, a relatively quick but structured intervention may be able to positively change the patient's lifestyle behavior. While these results are encouraging, they should be interpreted with caution, and further studies with larger sample sizes or longer follow‐up are needed to confirm these findings.

Our investigation did not detect any significant positive effect of either recommendation alone or recommendation plus guidebook interventions on sedentary behaviors. This may probably be due to the fact that the advice and the guidebook were primarily developed to increase physical activity rather than reduce sedentary behaviors. In this sense, strategies to reduce sedentary behaviors involve approaches that limit the overall time spent in activities characterized by very low energy expenditure, such as standing instead of sitting when possible, taking breaks from screens [40]. We chose to focus on physical activity rather than sedentary lifestyles based on current evidence. Indeed, whereas the literature highlights the association between physical activity and survival, the evidence regarding sedentary behaviors appears controversial. For instance, a large cohort study involving 161,808 people and a mean follow‐up of 11.8 years did not observe any significant association between hours spent in sedentary activities and lung cancer incidence or mortality but revealed a 32% lower risk of lung cancer mortality in those reporting moderate levels of physical activity [41]. However, despite the lack of impact on the oncological disease course, it should be remembered that sedentary behaviors are recognized risk factors for other chronic non‐communicable conditions, such as cardiovascular and metabolic diseases [42], to which patients could be exposed. Future studies should consider incorporating specific instructions to reduce sedentary time, in addition to promoting physical activity.

Some improvements in cognitive and social functioning and in constipation were observed, whereas no significant changes were observed in other domains of QoL. These findings are in line with the prior investigations on patients with breast and colorectal cancer that observed improvements in a few areas of QoL and fatigue [30, 43] but do not mirror the current literature. Indeed, several reviews and meta‐analyses reported the positive impact of physical activity on functioning and symptoms domains of QoL, and its effect is so robust that, for example, exercise has been indicated as a first‐line treatment for fatigue management [44]. Among the possible reasons for the lack of effect registered in our study could be the time of exposure and the supervision of the intervention. The guidelines of the American College of Sports Medicine suggest that, to be effective in enhancing physical fitness and QoL and in reducing symptoms, an exercise intervention should last at least 12 weeks [24]. In this perspective, it is possible that our intervention did not reveal significant changes because of the latency time required to observe improvements in QoL in response to physical activity. On the other hand, an individual patient data meta‐analysis of 34 randomized controlled trials found a significantly larger effect on QoL when the exercise was supervised by dedicated experts than the unsupervised intervention [45]. Considering these issues, in the future, it could be interesting to test interventions with a longer follow‐up time that also include a dedicated intervention (e.g., education) provided by exercise professionals, in addition to the oncologist's recommendation.

The study has some limitations that should be noted. First, we relied on self‐reported assessments of physical activity, which may have introduced reporting bias and imprecision. Second, the relatively short follow‐up period limits conclusions regarding long‐term adherence, sustainability of behavior change, and potential long‐term outcomes. Third, a potential selection bias should be considered, as patients who agreed to participate may have been more motivated or receptive to physical activity than the broader lung cancer population. At baseline, the groups showed slight differences in physical activity levels; however, this potential imbalance was accounted for in the generalized linear models by including baseline physical activity as a covariate. However, other potential confounders or sources of bias, such as symptom burden or comorbidities, may have influenced the results. An additional limitation of this study is the lack of detailed information regarding the specific types of exercise performed by participants and the individual barriers they may have encountered. Although patients with an ECOG performance status ≥ 2 were excluded, participants may still have experienced comorbid conditions such as musculoskeletal pain, balance impairment, or other physical limitations that could have influenced their ability to engage in certain forms or intensities of physical activity. Moreover, non‐physical barriers, including pre‐existing exercise habits, motivational factors, or competing life demands, may also have affected exercise participation. As such, a subset of participants may have benefited from a more targeted or individualized exercise support approach than that employed in the present study. However, data to further explore these aspects were not collected and should be addressed in future research.

Despite these limitations, this is the first study to investigate the effects of two brief, oncologist‐led exercise interventions in patients with lung cancer using a simple, easily implementable approach in routine clinical practice.

In summary, this randomized controlled trial showed that the oncologist's exercise recommendation can improve patients' physical activity level compared to usual care. Adding a dedicated guidebook further supported moderate‐intensity exercise engagement. These findings lay the foundation for incorporating brief, oncologist‐led exercise counseling into routine oncology practice and support the design of future hybrid interventions involving exercise professionals.

### 4.1 Implications for Dissemination and Scalability

The brevity and simplicity of the oncologist‐delivered recommendation represent major strengths that support its dissemination across different healthcare systems. In resource‐limited settings, where access to structured exercise programs or exercise professionals may be scarce, such a low‐cost, time‐efficient strategy could serve as a feasible first step to promote physical activity among patients with cancer. Conversely, in well‐resourced healthcare systems, oncologist‐based advice may serve as the initial entry point within a broader referral pathway involving physiotherapists, exercise specialists, or rehabilitation services. Importantly, the oncologists involved in our trial required only a 90‐min training session, which focused on reviewing the study protocol, summarizing current evidence on the benefits of exercise in oncology, and practicing the standardized delivery of the recommendation and guidebook. This concise training is easily replicable through short workshops or online modules, thereby supporting scalability and facilitating integration of brief exercise counseling into routine oncology care.

## Author Contributions

**Alice Avancini:** conceptualization (equal), data curation (equal), formal analysis (equal), funding acquisition (equal), investigation (equal), methodology (equal), project administration (equal), resources (equal), visualization (equal), writing – original draft (equal), writing – review and editing (equal). **Lorenzo Belluomini:** investigation (equal), resources (equal), visualization (equal), writing – review and editing (equal). **Diana Giannarelli:** formal analysis (equal), methodology (equal), visualization (equal), writing – review and editing (equal). **Jessica Insolda:** data curation (equal), resources (equal), visualization (equal), writing – review and editing (equal). **Anita Borsati:** visualization (equal), writing – review and editing (equal). **Marco Sposito:** resources (equal), visualization (equal), writing – review and editing (equal). **Jessica Menis:** resources (equal), visualization (equal), writing – review and editing (equal). **Paolo Lavagnolo:** resources (equal), visualization (equal), writing – review and editing (equal). **Daniela Tregnago:** visualization (equal), writing – review and editing (equal). **Ilaria Trestini:** visualization (equal), writing – review and editing (equal). **Federico Schena:** visualization (equal), writing – review and editing (equal). **Michele Milella:** visualization (equal), writing – review and editing (equal). **Sara Pilotto:** conceptualization (equal), data curation (equal), formal analysis (equal), funding acquisition (equal), investigation (equal), methodology (equal), project administration (equal), resources (equal), software (equal), visualization (equal), writing – original draft (equal), writing – review and editing (equal).

## Funding

The authors have nothing to report.

## Ethics Statement

All subjects provided informed written consent before enrollment in the study. The present study was approved by the ethics committee of the University of Verona (Prot. No. 26622), and registered on ClinicalTrial.gov.

## Conflicts of Interest

The authors declare no conflicts of interest.

## Supporting information


**Table S1:** Baseline physical exercise levels, sedentary behavior and quality of life of patients with lung cancer.
**Table S2:** Unadjusted values of the effect of the oncologist's recommendations on physical exercise levels in patients with lung cancer.
**Table S3:** Unadjusted values of the effect of the oncologist's recommendations on sedentary behavior and quality of life in patients with lung cancer.

## Data Availability

The data supporting the findings of this study are available within the article and/or its Supporting Information.
