# Supplementary material for: Impact of the Oncologist's Recommendation on Exercise Levels and Quality of Life in Patients With Lung Cancer: The ORE Randomized Controlled Trial
Source: Cancer Med. 2026 Apr 26;15(5):e71857. doi: 10.1002/cam4.71857 (PMC13111415; doi:10.1002/cam4.71857)
Supplement: Supplementary file 1 — Table S1: Baseline physical exercise levels, sedentary behavior and quality of life of patients with lung cancer. Table S2: Unadjusted values of the effect of the oncologist's recommendations on physical exercise levels in patients with lung cancer. Table S3: Unadjusted values of the effect of the oncologist's recommendations on sedentary behavior and quality of life in patients with lung cancer. [file CAM4-15-e71857-s001.docx]

**Supplementary Material**

**Table s1.** Baseline physical exercise levels, sedentary behaviour and quality of life of patients with lung cancer

**Table s2.** Unadjusted values of the effect of the oncologist’s recommendations on physical exercise levels in patients with lung cancer

**Table s3.** Unadjusted values of the effect of the oncologist’s recommendations on sedentary behaviour and quality of life in patients with lung cancer

**Table s1.** Baseline physical exercise levels, sedentary behaviour and quality of life of patients with lung cancer

| Characteristics | Total (n=91) | Control (n=29) | Exercise recommendation (n=31) | Exercise recommendation plus guidebook (n=31) | *p*-value |
| --- | --- | --- | --- | --- | --- |
| *Physical exercise (min/week)* |  |  |  |  |  |
| Total | 196.7 (433.4) | 45.0 (0.0-153.7)* | 210.0 (20.0-405.5)* | 90.0 (30.0-217.5)* | 0.07 |
| Vigorous | 0.7 (6.3) | 0.0 (0.0-0.0)* | 0.0 (0.0-0.0)* | 0.0 (0.0-0.0)* | 0.38 |
| Moderate | 22.2 (66.9) | 0.0 (0.0-0.0)* | 0.0 (0.0-0.0)* | 0.0 (0.0-0.0)* | 0.42 |
| Light | 173.9 (416.4) | 45.0 (0.0-123.7)* | 100.0 (6.7-277.5)* | 90.0 (16.2-202.5)* | 0.14 |
| *Sedentary behaviour (hours/day)* |  |  |  |  |  |
| Total (hours/week) | 14.7 (8.9) | 11.5 (5.9-15.6)* | 14.5 (10.7-29.7)* | 12.5 (9.7-15.9)* | 0.02 |
| Weekday | 6.3 (3.2) | 5.9 (3.6) | 5.4 (2.7) | 7.4 (3.0) | 0.04 |
| Weekend | 8.4 (8.5) | 5.5 (2.9-7.3)* | 7.3 (4.6-27.8)* | 5.5 (4.1-6.3)* | 0.03 |
| *Quality of life* |  |  |  |  |  |
| Physical function | 83.6 (18.5) | 83.2 (20.5) | 84.5 (17.3) | 83.2 (18.1) | 0.95 |
| Role function | 81.3 (26.7) | 82.8 (28.0) | 78.0 (29.9) | 83.3 (22.4) | 0.69 |
| Emotional function | 72.9 (25.5) | 74.1 (25.7) | 76.3 (22.2) | 68.3 (28.3) | 0.44 |
| Cognitive function | 89.9 (16.8) | 90.2 (21.1) | 88.7 (15.7) | 90.9 (13.5) | 0.88 |
| Social function | 84.1 (22.6) | 83.9 (24.2) | 84.9 (23.3) | 83.3 (21.1) | 0.96 |
| Global health status | 68.7 (23.2) | 63.2 (27.7) | 72.3 (19.2) | 70.2 (22.1) | 0.29 |
| Fatigue | 24.2 (25.4) | 25.7 (28.2) | 24.7 (23.1) | 22.2 (25.5) | 0.86 |
| Nausea and vomiting | 3.8 (10.8) | 3.4 (14.3) | 4.8 (9.8) | 3.2 (8.0) | 0.82 |
| Pain | 22.2 (25.3) | 23.0 (28.7) | 24.2 (23.1) | 19.3 (24.8) | 0.74 |
| Dyspnea | 27.1 (28.1) | 27.6 (26.8) | 23.7 (30.1) | 30.1 (27.7) | 0.67 |
| Insomnia | 23.8 (29.9) | 27.6 (30.9) | 20.4 (28.1) | 23.7 (31.3) | 0.66 |
| Appetite loss | 12.4 (24.7) | 11.5 (25.6) | 12.9 (25.3) | 12.9 (23.8) | 0.97 |
| Constipation | 18.7 (28.2) | 20.7 (30.1) | 16.1 (25.6) | 19.3 (29.5) | 0.81 |
| Diarrhea | 7.3 (18.5) | 5.7 (20.1) | 7.5 (16.6) | 8.6 (19.2) | 0.84 |
| Financial problems | 11.4 (22.3) | 11.5 (24.0) | 10.7 (24.9) | 11.8 (18.3) | 0.98 |
| *Data presented as median and interquartile range if normality test failed | | | | | |

**Table s2.** Unadjusted values of the effect of the oncologist’s recommendations on physical exercise levels in patients with lung cancer

|  | **Between group differences^§^** | | |
| --- | --- | --- | --- |
|  | **Exercise Recommendation vs Control** | **Exercise Recommendation plus guidebook vs Control** | **Exercise Recommendation vs Exercise Recommendation plus guidebook** |
|  | *Mean (95% CI)* | *Mean (95% CI)* | *Mean (95% CI)* |
| Total (min/week) |  |  |  |
| Baseline | +114.4  (+10.3; +218.4)* | +143.8  (-119.5; +407.1) | +29.4  (-230.5; +289.3) |
| Change from 0 to 4 weeks | -37.0  (-336.3; +292.2) | -284.5  (-596.3; +27.3) | -247.5  (-611.2; +116.2) |
| Change from 0 to 8 weeks | +110.4  (-108.0; +328.8) | -1.4  (-380.3; +377.5) | -111.8  (-525.7; +302.1) |
| Vigorous (min/week) |  |  |  |
| Baseline | +1.9  (-2.1; +5.9) | -- | -1.9  (-5.8; +1.9) |
| Change from 0 to 4 weeks | +10.7  (-12.1; +33.5) | +1.0  (-1.2; +3.3) | -9.7  (-30.9; +11.5) |
| Change from 0 to 8 weeks | +8.1  (-8.5; +24.6) | +12.0  (-14.4; +38.4) | +3.9  (-26.0; +33.9) |
| Moderate (min/week) |  |  |  |
| Baseline | +30.1  (-10.6; +70.7) | +0.7  (-16.8; +18.2) | -29.4  (-67.9; +9.2) |
| Change from 0 to 4 weeks | -48.0  (-101.7; +5.7) | +4.3  (-36.6; +45.1) | +52.3  (+1.8; +102.7)* |
| Change from 0 to 8 weeks | -24.2  (-81.7; +33.4) | +95.6  (-80.1; +271.3) | +119.8  (-56.5; +296.0) |
| Light (min/week) |  |  |  |
| Baseline | +82.4  (-7.6; +172.4) | +173.1  (-112.8; +399.0) | +60.7  (-190.2; +311.6) |
| Change from 0 to 4 weeks | +0.3  (-286.6; +287.2) | -289.8  (-595.4; +15.7) | -290.1  (-641.1; +60.9) |
| Change from 0 to 8 weeks | +164.9  (-57.4; +387.2) | -141.1  (-445.5; +163.3) | -306.0  (-659.2; +47.2) |
| ^§^ unadjusted values; * p<0.05 | | | |

**Table s3.** Unadjusted values of the effect of the oncologist’s recommendations on sedentary behaviour and quality of life in patients with lung cancer

|  | **Between group differences^§^** | | |
| --- | --- | --- | --- |
|  | **Exercise Recommendation vs Control** | **Exercise Recommendation plus guidebook vs Control** | **Exercise Recommendation vs Exercise Recommendation plus guidebook** |
|  | *Mean (95% CI)* | *Mean (95% CI)* | *Mean (95% CI)* |
| Sedentary total (hour/week) |  |  |  |
| Change from 0 to 4 weeks | 6.7  (-4.6; 17.9) | -4.5  (-17.1; 8.2) | -11.2  (-20.6; -1.7)* |
| Change from 0 to 8 weeks | 8.2  (-1.0; 17.3) | -1.0  (-12.0; 10.0) | -9.2  (-20.0; 1.7) |
| Sedentary weekday (hour/week) |  |  |  |
| Change from 0 to 4 weeks | 1.1  (-0.6; 2.9) | -0.6  (-2.6; 1.4) | -1.7  (-3.3; -0.1)* |
| Change from 0 to 8 weeks | 1.3  (-0.2; 2.8) | -0.4  (-2.2; 1.5) | -1.7  (-3.5; 0.1) |
| Sedentary weekend (hour/week) |  |  |  |
| Change from 0 to 4 weeks | 0.5  (-1.3; 2.3) | -0.7  (-2.5; 1.1) | -1.2  (-2.5; -0.1)* |
| Change from 0 to 8 weeks | 0.8  (-0.5; 2.0) | 0.4  (-1.3; 2.0) | -0.4  (-1.9; 1.1) |
| **Quality of life** | | | |
| Physical function |  |  |  |
| Change from 0 to 4 weeks | 1.7  (-6.7; 10.2) | 1.6  (-8.1; 11.3) | 0.1  (-8.1; 8.4) |
| Change from 0 to 8 weeks | -7.7  (-17.1; 1.8) | -4.4  (-15.6; 6.9) | -3.3  (-12.7; 6.1) |
| Role function |  |  |  |
| Change from 0 to 4 weeks | 1.3  (-16.3; 18.8) | -10.4  (-28.9; 8.1) | 11.7  (-3.2; 26.6) |
| Change from 0 to 8 weeks | -1.6  (-13.1; 10.0) | -10.2  (-24.5; 4.0) | 8.6  (-5.9; 23.1) |
| Emotional function |  |  |  |
| Change from 0 to 4 weeks | -6.2  (-16.5; 4.0) | 4.6  (-9.0; 18.2) | -10.8  (-23.1; 1.4) |
| Change from 0 to 8 weeks | -5.8  (-18.9; 7.2) | 0.8  (-14.5; 16.0) | -6.6  (-20.4; 7.1) |
| Cognitive function |  |  |  |
| Change from 0 to 4 weeks | -8.7  (-20.8; 3.3) | -11.6  (-20.5; -2.7)* | 2.9  (-7.5; 13.3) |
| Change from 0 to 8 weeks | -6.6  (-17.1; 3.9) | -6.3  (-14.9; 2.3) | -0.3  (-7.5; 7.0) |
| Social function |  |  |  |
| Change from 0 to 4 weeks | 3.8  (-9.3; 16.9) | -8.9  (-23.8; 6.0) | 12.7  (0.9; 24.5)* |
| Change from 0 to 8 weeks | -4.5  (-18.7; 9.6) | -6.9  (-19.3; 5.6) | 2.4  (-10.4; 15.1) |
| Global health status |  |  |  |
| Change from 0 to 4 weeks | 2.4  (-10.8; 15.6) | -2.6  (-15.1; 10.0) | 5.0  (-6.0; 16.0) |
| Change from 0 to 8 weeks | 1.7  (-13.5; 16.9) | -8.1  (-22.1; 5.9) | 9.8  (-4.1; 23.6) |
| Fatigue |  |  |  |
| Change from 0 to 4 weeks | -7.9  (-24.0; 8.2) | -3.2  (-15.7; 9.3) | 4.7  (-9.9; 19.3) |
| Change from 0 to 8 weeks | 11.0  (-5.5; 27.5) | -0.1  (-14.0; 13.9) | -11.1  (-24.5; 2.3) |
| Nausea and vomiting |  |  |  |
| Change from 0 to 4 weeks | 0.2  (-8.7; 9.1) | -1.0  (-9.1; 7.0) | -1.2  (-10.5; 8.2) |
| Change from 0 to 8 weeks | 1.1  (-6.6; 8.9) | -0.5  (-8.2; 7.1) | -1.7  (-10.1; 6.8) |
| Pain |  |  |  |
| Change from 0 to 4 weeks | 1.2  (-9.7; 12.1) | 2.1  (-10.9; 15.1) | 0.9  (-12.4; 14.2) |
| Change from 0 to 8 weeks | 9.4  (-2.4; 21.3) | 4.1  (-8.4; 16.6) | -5.3  (-19.4; 8.7) |
| Dyspnea |  |  |  |
| Change from 0 to 4 weeks | 5.5  (-11.0; 22.0) | 6.9  (-11.7; 25.4) | 1.4  (-16.0; 18.8) |
| Change from 0 to 8 weeks | 9.4  (-6.6; 25.4) | 5.2  (-10.8; 21.2) | -4.2  (-20.6; 12.1) |
| Insomnia |  |  |  |
| Change from 0 to 4 weeks | -3.4  (-18.1; 11.2) | 1.0  (-13.6; 15.6) | 4.4  (-14.4; 23.2) |
| Change from 0 to 8 weeks | 2.7  (-10.4; 15.7) | 6.7  (-6.9; 20.2) | 4.0  (-12.2; 20.2) |
| Appetite loss |  |  |  |
| Change from 0 to 4 weeks | -3.1  (-17.6; 11.3) | -9.4  (-25.7; 6.8) | -6.3  (-22.4; 9.8) |
| Change from 0 to 8 weeks | 0.1  (-15.0; 15.2) | -4.8  (-20.9; 11.3) | -4.9  (-22.1; 12.3) |
| Constipation |  |  |  |
| Change from 0 to 4 weeks | -3.9  (-16.2; 8.5) | -6.9  (-20.2; 6.4) | -3.1  (-19.1; 12.9) |
| Change from 0 to 8 weeks | 17.2  (0.7; 33.7)* | 0.3  (-13.4; 14.1) | -16.9  (-33.0; -0.8)* |
| Diarrhea |  |  |  |
| Change from 0 to 4 weeks | 0.1  (-13.5; 13.8) | 2.3  (-10.3; 14.8) | 2.1  (-14.2; 18.5) |
| Change from 0 to 8 weeks | 2.7  (-9.3; 14.6) | -5.6  (-17.5; 6.4) | -8.2  (-20.4; 4.0) |
| Financial problems |  |  |  |
| Change from 0 to 4 weeks | 0.8  (-12.4; 13.9) | -6.7  (-20.2; 6.8) | -7.5  (-22.5; 7.5) |
| Change from 0 to 8 weeks | -0.1  (-13.7; 13.5) | -1.9  (-15.1; 11.3) | -1.8  (-14.8; 11.2) |
| ^§^ unadjusted values; *p<0.05 | | | |
